# Towards Rapid Bedside Detection of Sarcopenia in Cancer Patients: The Role of Rectus Femoris Muscle Ultrasonography—A Prospective Cross-Sectional Study

**DOI:** 10.3390/medicina62020413

**Published:** 2026-02-21

**Authors:** Süleyman Baş, Hasan Hakan Çoban, Akif Doğan, Hande Nur Erölmez, Hasan Hüseyin Mutlu, Nurullah İlhan

**Affiliations:** 1 Department of Internal Medicine, University of Health Sciences, Sancaktepe Sehit Prof. Dr. Ilhan Varank Training and Research Hospital, 34785 Istanbul, Turkey; cobanhhakan@gmail.com; 2 Department of Medical Oncology, University of Health Sciences, Sancaktepe Sehit Prof. Dr. İlhan Varank Training and Research Hospital, 34785 Istanbul, Turkey; drakifd@gmail.com (A.D.);; 3 Department of Family Medicine, University of Health Sciences, Sancaktepe Sehit Prof. Dr. İlhan Varank Training and Research Hospital, 34785 Istanbul, Turkey; herolmez@gmail.com (H.N.E.);

**Keywords:** sarcopenia, rectus femoris, handgrip strength, bioelectrical impedance analysis

## Abstract

*Background and Objectives*: Sarcopenia is a common yet underrecognized condition in cancer patients and is associated with increased treatment-related toxicity, functional decline, and poor clinical outcomes. Practical, rapid, and bedside-applicable tools are needed to detect sarcopenia early in routine oncology practice. This study aimed to evaluate the diagnostic value of rectus femoris muscle ultrasonography within an integrated clinical assessment combining handgrip strength and bioelectrical impedance analysis for the detection of sarcopenia in cancer patients. *Materials and Methods*: In this prospective cross-sectional study, 147 adult patients with malignancy were evaluated using a multimodal sarcopenia assessment framework. Muscle strength was assessed by handgrip dynamometry, muscle mass was estimated using bioelectrical impedance analysis (BIA)-derived appendicular skeletal muscle mass index (ASMI), and muscle morphology was evaluated using ultrasonographic measurements of the rectus femoris and biceps brachii muscles. Sarcopenia was defined and classified according to the EWGSOP2 criteria. Associations between clinical variables, BIA parameters, and ultrasonographic measurements were analyzed. Receiver operating characteristic (ROC) curve analyses were performed to assess the diagnostic performance of muscle ultrasonography for sarcopenia detection. *Results*: The mean age of the study population was 60.2 ± 11.2 years, and 51% of participants were male. Confirmed sarcopenia was identified in 12.2% of patients, while 27.2% were classified as having probable sarcopenia. Sarcopenic patients were significantly older (68.5 ± 7.6 vs. 59.0 ± 11.2 years, *p* = 0.001) and had lower handgrip strength (15.8 ± 6.0 vs. 24.3 ± 8.4 kg, *p* < 0.001) and ASMI values (5.96 ± 0.64 vs. 7.23 ± 1.18 kg/m^2^, *p* < 0.001). Rectus femoris muscle thickness was significantly reduced in patients with sarcopenia (6.40 ± 1.42 vs. 8.19 ± 2.21 mm, *p* = 0.001). Rectus femoris muscle thickness demonstrated good diagnostic performance for sarcopenia detection (AUC = 0.752; 95% CI: 0.650–0.853; *p* = 0.001), with an optimal cut-off value of ≤7.59 mm (sensitivity 83.3%, specificity 61.2%). *Conclusions*: Rectus femoris muscle ultrasonography is a practical, rapid bedside assessment for detecting sarcopenia in cancer patients. When integrated with handgrip strength and BIA, this multimodal approach provides a feasible, radiation-free strategy for early sarcopenia screening in routine oncology practice.

## 1. Introduction

Sarcopenia is a condition characterized by a progressive decline in skeletal muscle mass, muscle strength, and physical performance, and it is strongly associated with adverse clinical outcomes. Although traditionally considered an age-related phenomenon, sarcopenia is now recognized to be closely linked to malignancy, chronic systemic inflammation, inadequate nutritional intake, and cancer-related metabolic disturbances [1,2]. In patients with cancer, sarcopenia has been associated with a broad spectrum of adverse clinical outcomes, including postoperative complications, functional decline, reduced survival, and an overall increase in treatment-related toxicity, as consistently demonstrated in large-scale systematic reviews and meta-analyses [3,4,5,6]. Specifically among patients receiving chemotherapy, sarcopenia has been linked to a higher risk of chemotherapy-related toxicity, which may result in dose reductions, treatment delays, or treatment discontinuation, as well as a greater incidence of severe treatment-related adverse events [7]. Collectively, these findings highlight sarcopenia not merely as a comorbid condition but as a potentially modifiable risk factor that directly influences treatment tolerance and clinical outcomes.

Early identification and accurate assessment of sarcopenia are therefore essential for implementing nutritional interventions, exercise strategies, and individualized treatment planning. Nevertheless, sarcopenia frequently remains underrecognized in routine clinical practice. Conventional anthropometric measures, such as body mass index, are insufficient because they fail to differentiate between lean tissue and fat mass [8]. Although computed tomography (CT) and magnetic resonance imaging (MRI) are considered the gold-standard methods for assessing skeletal muscle mass, their routine use for screening purposes is limited by radiation exposure, high cost, and restricted accessibility [9,10]. Consequently, there is a clear need for rapid, reliable, and clinically feasible alternative assessment methods in oncology settings.

Bioelectrical impedance analysis (BIA) is a widely used, non-invasive, and portable method for evaluating body composition in clinical practice. Both European and Asian sarcopenia consensus guidelines support the use of BIA for estimating muscle mass [11]. In oncology populations, low muscle mass, as measured by BIA, has been associated with poor clinical outcomes and increased mortality [12]. However, BIA measurements may be influenced by factors such as hydration status, edema, and ascites, and these limitations should be carefully considered when interpreting results.

In recent years, muscle ultrasonography has emerged as a promising tool for assessing sarcopenia. By allowing direct visualization and measurement of muscle thickness and cross-sectional area, ultrasonography provides real-time morphological information without radiation exposure. Its bedside applicability, repeatability, and user-friendly nature make it particularly attractive for integration into oncology practice [13,14]. Ultrasonographic measurements of the rectus femoris muscle, in particular, have demonstrated strong correlations with overall skeletal muscle mass and functional capacity [15]. Accumulating evidence suggests that muscle ultrasonography may achieve diagnostic performance comparable to BIA- and CT-based assessments and may offer greater sensitivity in selected patient populations [16,17]. Accordingly, muscle ultrasonography holds considerable potential for broader application in the diagnosis and management of sarcopenia.

In this context, further studies are needed to evaluate the clinical utility of BIA and muscle ultrasonography for identifying sarcopenia in patients with cancer. The rectus femoris was selected as a representative lower-limb muscle for ultrasonographic assessment due to its established role in sarcopenia research and its strong association with mobility and functional performance. Quadriceps muscles, particularly the rectus femoris, have been widely used in ultrasound-based sarcopenia assessment protocols and have demonstrated correlations with whole-body muscle mass and clinical outcomes [13,14].

In addition, the biceps brachii was included to enable assessment of upper-limb muscle morphology and to explore potential regional differences in muscle loss. Previous studies have shown that upper-extremity muscle thickness correlates with appendicular lean mass and muscle strength, supporting its use in sarcopenia research [17,18]. Evaluating both lower- and upper-extremity muscles may provide a more comprehensive representation of appendicular muscle involvement, particularly in oncology populations where muscle wasting may be heterogeneous.

The primary objective of the present study was to evaluate the diagnostic performance of rectus femoris muscle ultrasonography for detecting sarcopenia in an oncology population. Secondary objectives included examining the associations between ultrasonographic measurements of both the rectus femoris and biceps brachii, BIA-derived skeletal muscle parameters, and handgrip strength, as well as assessing the practical applicability of these radiation-free modalities in routine oncology practice.

## 2. Materials and Methods

### 2.1. Ethical Approval and Participant Rights

The study was approved by the Clinical Research Ethics Committee of Sancaktepe Şehit Prof. Dr. İlhan Varank Training and Research Hospital (Decision No: E-46059653-050.04-273314831, Date: 11 April 2025). All procedures were conducted in full accordance with the ethical principles of the Declaration of Helsinki (1975). All participants received detailed verbal and written information about the study, and written informed consent was obtained before enrollment.

### 2.2. Study Design and Participants

This prospective cross-sectional study was conducted at the Medical Oncology Outpatient Clinic of Sancaktepe Şehit Prof. Dr. İlhan Varank Training and Research Hospital between 3 April 2025 and 1 October 2025. Adult patients (≥18 years) with a malignancy diagnosis who were under regular follow-up or receiving active systemic antineoplastic treatment were eligible for inclusion. All clinical assessments and measurements were performed consecutively on the same day during routine outpatient visits to ensure data standardization. All eligible patients who met the inclusion criteria during the study period were consecutively enrolled. No a priori sample size or power calculation was performed, as the study was designed as an exploratory, real-world observational investigation.

### 2.3. Inclusion and Exclusion Criteria

Patients who were able to comply with the predefined measurement protocol (handgrip dynamometry, BIA, and ultrasonography) and who provided written informed consent were included.

To maintain data reliability and measurement accuracy, patients meeting any of the following criteria were excluded:

Functional and Neuromuscular Limitations: Primary neuromuscular disorders that could directly affect muscle strength or mass assessment (e.g., paraplegia, quadriplegia), severe hand osteoarthritis, advanced peripheral neuropathy, or peripheral arterial disease.

Technical and Physical Barriers: Presence of implanted electronic devices that could interfere with BIA measurements (e.g., pacemakers, implantable cardioverter-defibrillators), limb amputation, joint contractures, or a history of active fracture involving the hand, foot, or lower extremity at the time of assessment.

Fluid Balance Disorders: Conditions that could distort tissue imaging or increase measurement error in BIA and ultrasonographic assessments, including severe peripheral edema, generalized anasarca, clinically significant ascites, or pleural effusion.

Clinical and Cognitive Conditions: Severe cognitive impairment or neuropsychiatric disorders that could prevent compliance with measurement instructions (particularly during handgrip testing), pregnancy, and poor general condition precluding tolerance of the assessment procedures (Eastern Cooperative Oncology Group performance status ≥ 3).

**Figure 1:**
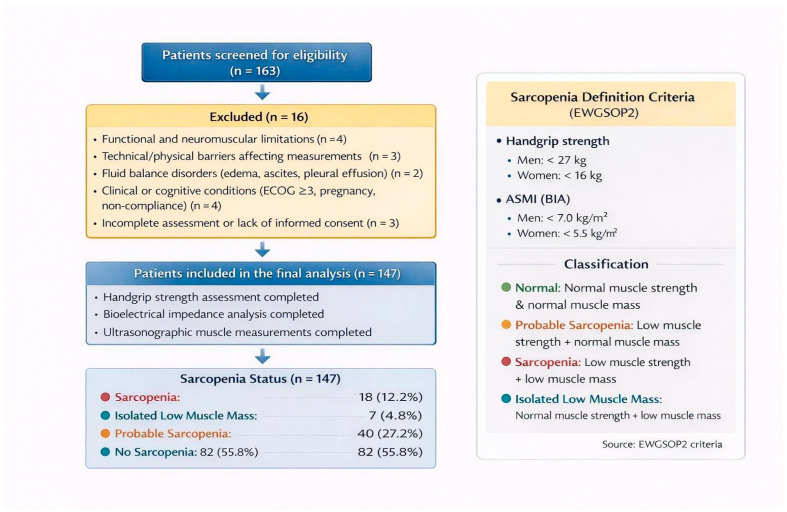
Patient Flow Diagram and Classification of Sarcopenia According to EWGSOP2 Criteria.

Based on these criteria, 147 patients with complete datasets were included in the final analysis (Figure 1).

### 2.4. Data Collection and Clinical Assessments

Demographic data (age, sex) and detailed clinical characteristics (cancer type, TNM stage, time since diagnosis, comorbidities, and current oncologic treatments) were obtained from the hospital’s electronic medical record system and archived patient files, supplemented by face-to-face interviews using standardized case report forms. Comorbidities were recorded as documented chronic conditions based on medical records; however, a formal comorbidity index (e.g., Charlson Comorbidity Index) was not applied. Height and body weight were measured for all participants, and body mass index (BMI) was calculated as kg/m^2^. A formal nutritional assessment using standardized screening tools (e.g., MNA, NRS-2002, or MUST) was not performed. Following anthropometric assessments, each participant underwent sequential evaluation of sarcopenia components: handgrip strength assessment for muscle function, bioelectrical impedance analysis for body composition and total muscle mass, and ultrasonographic measurements of the rectus femoris and biceps brachii muscles to quantitatively assess muscle morphology (thickness and cross-sectional area).

### 2.5. Assessment of Muscle Strength (Handgrip Strength)

Muscle strength was assessed using a calibrated hydraulic handgrip dynamometer (Baseline^®^ Hydraulic Hand Dynamometer, New York, NY, USA). Measurements were performed in accordance with the standardized testing position recommended by the American Society of Hand Therapists (ASHT) [19]. Participants were seated comfortably with the shoulder adducted, the elbow flexed at 90°, the forearm in a neutral position, and the wrist maintained in a neutral position. Measurements were obtained from the dominant hand. To minimize fatigue, three maximal isometric contractions were performed with one-minute rest intervals between trials. Standardized verbal encouragement was provided during each attempt to ensure maximal effort. The mean value of the three measurements was recorded in kilograms (kg) and used for statistical analyses. This approach was preferred to minimize the impact of potential outliers and trial-to-trial variability, thereby providing a more stable estimate of maximal voluntary muscle strength in this oncology population. Handgrip strength was selected as the primary indicator of muscle function, in line with the recommendations of the European Working Group on Sarcopenia in Older People 2 (EWGSOP2), which defines reduced muscle strength as the key feature of probable sarcopenia [1].

### 2.6. Body Composition Analysis (Bioelectrical Impedance Analysis)

Body composition was assessed using a single-frequency bioelectrical impedance analysis (BIA) device (Tanita SC-330MA, Tanita Corporation, Tokyo, Japan). To minimize measurement variability, all assessments were conducted according to a standardized protocol [8]. Measurements were performed in the morning following an overnight fast. Participants were instructed to refrain from food intake for at least 8 h, avoid vigorous physical activity for 24 h, and abstain from alcohol and caffeine for at least 12 h before the assessment. Fluid intake was restricted for 2–3 h before measurement, and participants were asked to empty their bladders immediately beforehand. After removal of all metal accessories, measurements were obtained barefoot on a flat surface to ensure adequate electrode contact, as recommended by established guidelines [8].

Participants stood upright on the device with their arms slightly abducted and remained motionless during the assessment. Height, body weight, and bioelectrical impedance values were automatically recorded. Skeletal muscle mass (SMM) was estimated using the equation proposed by Janssen et al., which has been validated against magnetic resonance imaging (MRI) [20]:

SMM (kg) = ((Height^2^/R) × 0.401) + (Sex × 3.825) + (Age × −0.071) + 5.102, where height is expressed in centimeters, R represents resistance in ohms, sex is coded as 1 for men and 0 for women, and age is expressed in years.

To standardize muscle mass for body size, the appendicular skeletal muscle mass index (ASMI) was estimated based on previously reported skeletal muscle distribution data suggesting that appendicular muscle mass represents approximately 75% of total skeletal muscle mass [21,22,23]. Accordingly, ASMI was calculated as: ASMI (kg/m^2^) = (SMM × 0.75)/height^2^(m^2^). Low muscle mass was defined using EWGSOP2-recommended cut-off values (<7.0 kg/m^2^ for men and <5.5 kg/m^2^ for women) [1].

### 2.7. Ultrasonographic Muscle Assessment

Muscle ultrasonography was performed using a wireless, handheld ultrasound system (Model: WLPCCFB025; Sonostar Technologies, Guangzhou, China) equipped with a high-frequency (7.5–10 MHz) linear-array transducer. Imaging was displayed on a portable laptop via a dedicated wireless connection. To minimize inter-observer variability and ensure measurement standardization, all examinations were conducted by a single investigator who was formally trained and certified in musculoskeletal ultrasonography. Participants remained in the supine position for at least 5 min before imaging to allow adequate fluid redistribution. All assessments were performed in B-mode following standardized imaging protocols previously described in methodological studies [13]. Gain and depth settings were standardized and kept constant across all participants according to the predefined imaging protocol. To avoid muscle deformation, a generous amount of coupling gel was applied, and the transducer was positioned perpendicular to the muscle with minimal pressure. The rectus femoris was selected as a representative lower-limb muscle due to its established use in ultrasound-based sarcopenia assessment and its strong association with mobility and functional outcomes. The biceps brachii was included to assess upper-limb muscle status, enabling evaluation of regional muscle distribution and appendicular involvement in sarcopenia.

Rectus Femoris Measurements: Participants were evaluated in the supine position with both lower extremities fully extended and relaxed to minimize gravitational and postural influences on quadriceps morphology during ultrasound assessment, consistent with commonly used ultrasonographic sarcopenia protocols. The measurement site was defined as the midpoint between the anterior superior iliac spine and the superior border of the patella, as described in standardized protocols such as the ECOSARC study [24]. Quadriceps cross-sectional area measurements were performed in line with validation studies using wide field-of-view ultrasound systems [25]. Rectus femoris muscle thickness (RF-MT) and cross-sectional area (RF-CSA) were measured on B-mode images. Each parameter was assessed three times consecutively during the same session, and the mean value was used for statistical analysis.

Biceps Brachii Measurements: Biceps brachii thickness was measured with participants in a seated position, with the arm relaxed and supported to prevent involuntary muscle activation. The measurement site was standardized at the midpoint between the acromion and the antecubital fossa in accordance with recommended anatomical landmark–based ultrasound protocols for muscle assessment. Biceps brachii muscle thickness and cross-sectional area were measured using the same standardized transverse B-mode technique. Muscle thickness was defined as the perpendicular distance between the superficial and deep aponeuroses, and cross-sectional area was calculated using the system’s built-in area measurement function [14,26].

All ultrasound images were acquired using consistent device settings and digitally stored. To ensure reliability and reproducibility, all ultrasonographic assessments were performed by the same investigator following a predefined study protocol [13,17]. Echo intensity analysis was not performed, as the study focused primarily on structural muscle morphology parameters (thickness and cross-sectional area).

### 2.8. Definition and Classification of Sarcopenia

Sarcopenia was defined according to the diagnostic algorithm and cut-off values proposed in the revised EWGSOP2 consensus report (Figure 1). According to this framework, low muscle strength was considered indicative of probable sarcopenia, whereas low muscle mass was required to confirm the diagnosis [1].

Diagnostic Parameters and Cut-Off Values:

Muscle Strength: Low muscle strength was defined as handgrip strength <27 kg for men and <16 kg for women [1].

Muscle Mass: Low muscle mass was defined as ASMI values <7.0 kg/m^2^ in men and <5.5 kg/m^2^ in women, as measured by BIA [1].

### 2.9. Participant Classification

Based on the combined assessment of muscle strength and muscle mass, participants were classified into four categories:

Normal: Both muscle strength and muscle mass are above the established cut-off values.

Probable Sarcopenia: Reduced muscle strength with preserved muscle mass.

Sarcopenia: Concurrent reduction in both muscle strength and muscle mass.

Isolated Low Muscle Mass: Reduced muscle mass in the presence of preserved muscle strength.

This classification approach is fully aligned with EWGSOP2 and international guidelines, allowing objective identification of both early functional impairment and established sarcopenia [1]. Severe sarcopenia was not evaluated in this study, as physical performance assessments (e.g., gait speed or chair stand test) were not performed. For the purpose of statistical analyses, participants were dichotomized into sarcopenia and non-sarcopenia groups.

### 2.10. Statistical Analysis

Statistical analyses were conducted using IBM SPSS Statistics software (version 26.0; IBM Corp., Armonk, NY, USA). The distribution of continuous variables was assessed using the Shapiro–Wilk test. Variables with a normal distribution were summarized as mean ± standard deviation, while non-normally distributed variables were reported as median with interquartile range (IQR). Categorical variables were presented as frequencies and percentages.

Comparisons between patients with and without sarcopenia were performed according to data distribution. The independent-samples *t*-test was used for normally distributed continuous variables, and the Mann–Whitney U test was applied for non-normally distributed continuous variables. Categorical variables were compared using the chi-square test or Fisher’s exact test, as appropriate.

Associations between handgrip strength, parameters derived from bioelectrical impedance analysis, and ultrasonographic muscle measurements were examined using correlation analyses. Pearson correlation coefficients were applied for normally distributed variables, whereas Spearman rank correlation coefficients were used when normality assumptions were not met.

To identify factors independently associated with sarcopenia, univariable logistic regression analyses were initially performed. Variables with *p*-values < 0.10, together with clinically relevant covariates, were subsequently included in multivariable logistic regression models. To minimize multicollinearity, separate models were constructed for RF-MT and RF-CSA. Results were expressed as odds ratios (ORs) with corresponding 95% confidence intervals (CIs). Model calibration was assessed using the Hosmer–Lemeshow goodness-of-fit test. Given the limited number of sarcopenia events in the study population, the number of variables included in multivariable models was deliberately restricted to reduce the risk of overfitting and unstable estimates. Therefore, parsimonious models were constructed, including clinically relevant covariates (age and sex) alongside one ultrasonographic parameter per model.

Receiver operating characteristic (ROC) curve analyses were performed to evaluate the diagnostic performance of ultrasonographic parameters in identifying sarcopenia. The area under the ROC curve (AUC) was calculated, and optimal cut-off values were determined using the Youden index. Sensitivity and specificity corresponding to each cut-off value were reported. All statistical tests were two-sided, and *p*-values < 0.05 were considered statistically significant.

## 3. Results

### 3.1. Participant Characteristics

A total of 147 patients with malignancy were included in the study. The mean age was 60.18 ± 11.21 years, with males accounting for 51% of the study population. Mean height was 161.99 ± 9.35 cm, mean body weight was 69.62 ± 12.66 kg, and the mean body mass index (BMI) was 26.37 ± 5.50 kg/m^2^. Breast and gynecological cancers constituted the most frequent malignancy group (36.7%), followed by gastrointestinal cancers (30.6%). The median time since cancer diagnosis was 154 days (IQR: 58.75–462.75). Metastatic disease was present in 17.7% of patients, while 56.5% had at least one documented comorbidity. Detailed baseline demographic and clinical characteristics are presented in Table 1.

### 3.2. Muscle Strength and Body Composition

The mean handgrip strength of the study population was 23.28 ± 8.58 kg. Reduced muscle strength, corresponding to probable sarcopenia according to EWGSOP2 criteria, was observed in 39.5% of patients. Bioelectrical impedance analysis demonstrated a mean total muscle mass of 46.45 ± 7.57 kg, a mean skeletal muscle mass (SMM) of 24.82 ± 4.87 kg, and a mean appendicular skeletal muscle mass index (ASMI) of 7.08 ± 1.20 kg/m^2^. Based on the combined evaluation of muscle strength and muscle mass, 12.2% of patients were classified as having confirmed sarcopenia, 27.2% as probable sarcopenia, and 4.8% as isolated low muscle mass. Muscle strength and body composition parameters are summarized in Table 2.

### 3.3. Ultrasonographic Muscle Measurements Results

Ultrasonographic evaluation revealed a mean RF-MT of 7.98 ± 2.20 mm and a mean RF-CSA of 2.03 ± 0.60 cm^2^. For the upper extremity, mean biceps brachii muscle thickness was 8.80 ± 2.33 mm, and the median cross-sectional area was 2.15 [1.61–2.65] cm^2^. A detailed summary of ultrasonographic parameters is provided in Table 3.

### 3.4. Comparison Between Sarcopenia and Non-Sarcopenia Groups

Patients classified as having sarcopenia were significantly older than those without sarcopenia (68.50 ± 7.64 vs. 59.02 ± 11.16 years; *p* = 0.001) and were more frequently male (*p* = 0.001). In addition, the distribution of cancer types differed significantly between groups (*p* = 0.003), with a higher prevalence of sarcopenia observed among patients with respiratory system malignancies. All bioelectrical impedance analysis–derived muscle parameters were significantly lower in the sarcopenia group, including total muscle mass (42.81 ± 4.29 vs. 46.96 ± 7.80 kg; *p* = 0.002), skeletal muscle mass (21.81 ± 3.20 vs. 25.24 ± 4.93 kg; *p* < 0.001), and ASMI (5.96 ± 0.64 vs. 7.23 ± 1.18 kg/m^2^; *p* < 0.001). Handgrip strength was also markedly reduced in patients with sarcopenia (15.79 ± 6.01 vs. 24.32 ± 8.38 kg; *p* < 0.001), and all sarcopenic patients fulfilled the criteria for low muscle strength. Group comparisons are summarized in Table 4. RF-MT and RF-CSA were significantly lower in patients with sarcopenia than in those without (*p* = 0.001 and *p* = 0.003, respectively). Similarly, biceps brachii muscle thickness and cross-sectional area did not differ significantly between sarcopenic and non-sarcopenic patients. These findings are presented in Table 5.

### 3.5. Correlation Analyses

Handgrip strength demonstrated significant positive correlations with RF-MT (r = 0.371, *p* < 0.001) and RF-CSA (r = 0.414, *p* < 0.001). Appendicular skeletal muscle mass index was also positively correlated with rectus femoris muscle thickness (r = 0.195, *p* = 0.018) and RF-CSA (r = 0.180, *p* = 0.029). In addition, a weak but statistically significant positive correlation was observed between ASMI and biceps brachii cross-sectional area (r = 0.172, *p* = 0.038). Correlation results are detailed in Table 6.

### 3.6. Factors Associated with Sarcopenia

Multivariable logistic regression analyses identified age, male sex, and ultrasonographic parameters of the rectus femoris muscle as independent factors associated with sarcopenia. In Model 1, each one-year increase in age was associated with a 7.1% increase in the odds of sarcopenia (OR = 1.071; 95% CI: 1.002–1.143; *p* = 0.042), while male sex was associated with a substantially higher risk (OR = 9.313; 95% CI: 1.926–45.036; *p* = 0.006). Decreased rectus femoris muscle thickness remained independently associated with sarcopenia (OR = 0.613; 95% CI: 0.436–0.862; *p* = 0.005) in Table 7.

In Model 2, age (OR = 1.081; 95% CI: 1.013–1.154; *p* = 0.019) and male sex (OR = 9.056; 95% CI: 1.885–43.504; *p* = 0.006) remained significant predictors of sarcopenia. A reduction in RF-CSA was also independently associated with sarcopenia (OR = 0.320; 95% CI: 0.110–0.929; *p* = 0.036). Hosmer–Lemeshow goodness-of-fit tests indicated adequate model calibration for both models (Model 1: *p* = 0.922; Model 2: *p* = 0.867). Regression results are summarized in Table 7.

### 3.7. Diagnostic Performance of Ultrasonographic Measurements

**Figure 2:**
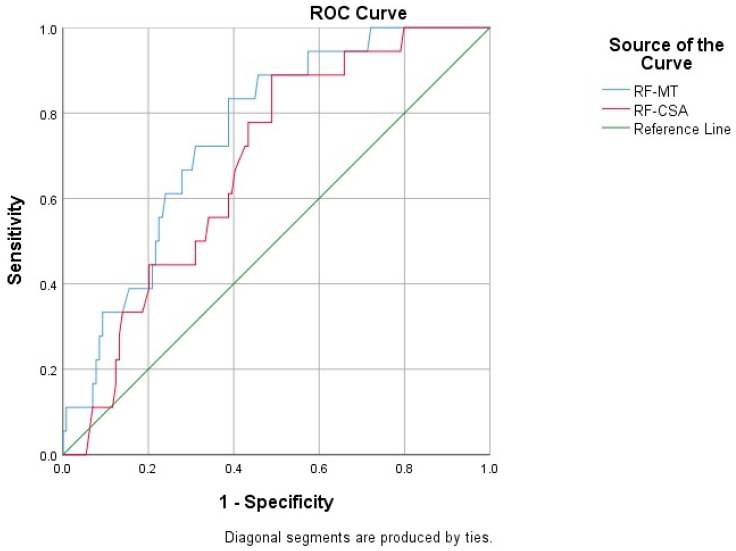
ROC curves of ultrasonographic parameters for predicting sarcopenia.

Receiver operating characteristic (ROC) curve analysis was performed to assess the diagnostic accuracy of ultrasonographic parameters in identifying sarcopenia (Figure 2). Rectus femoris muscle thickness demonstrated good discriminative ability, with an area under the curve (AUC) of 0.752 (95% CI: 0.650–0.853; *p* = 0.001). The optimal cut-off value for RF-MT was ≤7.59 mm, with a sensitivity of 83.3% and a specificity of 61.2%.

RF-CSA showed moderate diagnostic performance, with an AUC of 0.681 (95% CI: 0.571–0.791). An optimal cut-off value of ≤2.07 cm^2^ yielded a sensitivity of 88.9% and a specificity of 51.2%. Detailed ROC analysis results are presented in Table 8. ROC analysis was not performed for biceps brachii variables, as these parameters did not demonstrate statistically significant discriminatory capacity between sarcopenia and non-sarcopenia groups in the initial comparative analyses.

## 4. Discussion

In this prospective cross-sectional study, sarcopenia in patients with cancer was evaluated using a multimodal approach integrating handgrip strength, bioelectrical impedance analysis (BIA), and ultrasonographic muscle measurements. The main findings indicate that both sarcopenia and probable sarcopenia are common among oncology outpatients. In addition, lower-extremity ultrasonographic parameters (rectus femoris) showed a strong association with sarcopenia, whereas upper-extremity (biceps brachii) measurements did not. RF-MT and RF-CSA also demonstrated high diagnostic performance for predicting sarcopenia, independent of age and sex.

In our cohort, the prevalence of confirmed sarcopenia was 12.2%, while 27.2% of patients were classified as having probable sarcopenia according to EWGSOP2 criteria. Reported prevalence rates in cancer populations vary widely depending on tumor type, disease stage, and the diagnostic criteria used [4,27,28]. The relatively modest prevalence of confirmed sarcopenia in our cohort may partly be explained by the exclusion of patients with poor general condition (ECOG ≥ 3), which may have resulted in underrepresentation of more functionally impaired individuals. Notably, the high rate of probable sarcopenia observed in our study supports the concept that functional decline (reduced muscle strength) may precede overt structural loss of muscle mass (atrophy) [29,30]. This finding highlights the clinical importance of early functional screening in oncology practice beyond routine radiological imaging.

Handgrip strength was significantly lower in patients with sarcopenia. Moreover, handgrip strength showed positive correlations with both BIA-derived skeletal muscle mass (SMM) and appendicular skeletal muscle mass index (ASMI), as well as with ultrasonographic measurements of the rectus femoris muscle. These results are consistent with previous studies reporting that handgrip strength is a critical indicator of functional capacity and fatigue in patients with cancer [31]. Given the high prevalence of cancer-related fatigue and physical deconditioning during systemic therapy, handgrip strength assessment represents a rapid, practical, and clinically valuable tool that complements structural evaluations of muscle mass.

BIA-derived ASMI was significantly reduced in sarcopenic patients, emphasizing the utility of BIA as a practical method for estimating muscle mass in oncology settings. However, it is well recognized that BIA measurements—particularly in patients with advanced disease—can be influenced by hydration status, edema, and fluid retention [4,32]. These methodological limitations underscore the importance of incorporating additional assessment modalities that are less susceptible to fluid-related factors and allow direct visualization of muscle.

A notable finding of this study is the strong relationship between ultrasonographic measurements of the rectus femoris muscle and sarcopenia. Rectus femoris muscle thickness, cross-sectional area, and circumference were all significantly reduced in sarcopenic patients. In contrast, biceps brachii measurements did not differ significantly between groups. This pattern is consistent with previous evidence suggesting that lower-extremity muscles may serve as more sensitive indicators of systemic muscle loss than upper-extremity muscles [17,18]. The absence of significant differences in biceps brachii measurements may imply that upper-extremity muscles are relatively preserved during the earlier stages of sarcopenia. By contrast, the rectus femoris—an antigravity muscle group—plays a critical role in mobility, balance, and functional independence and may therefore be more vulnerable to inactivity, inflammation, and cancer-related catabolic processes. Previous ultrasound-based studies likewise reported that rectus femoris parameters are more effective than upper-limb measurements for detecting sarcopenia and functional impairment [17,18]. Furthermore, intramuscular fat infiltration (myosteatosis), which simple thickness measures may not fully capture, is associated with reduced muscle quality and may affect different muscle groups to varying degrees [33].

Multivariable logistic regression analyses identified age and male sex as independent predictors of sarcopenia, consistent with established epidemiological data [29,34,35,36]. Importantly, rectus femoris ultrasound parameters remained significantly associated with sarcopenia even after adjustment for these demographic factors. This finding indicates that muscle ultrasound provides valuable clinical information beyond traditional risk factors, supporting its role as a predictive assessment tool rather than merely a descriptive measure. However, the imbalance in metastatic disease distribution between groups (lower proportion in the sarcopenic group) may have influenced comparisons, as disease burden can affect muscle mass, systemic inflammation, and functional status. This heterogeneity should be considered when interpreting group differences.

ROC curve analysis strongly supported the diagnostic value of ultrasonographic evaluation of the rectus femoris muscle. Rectus femoris muscle thickness demonstrated good discriminative performance, with an area under the curve (AUC) of 0.752 and an optimal cut-off value of ≤7.59 mm. This level of diagnostic accuracy is consistent with prior sarcopenia studies and highlights the clinical relevance of rectus femoris cut-off values for screening. However, no universally accepted ultrasonographic cut-off values currently exist, and previously reported thresholds vary depending on population characteristics, measurement protocols, and anatomical landmarks. In geriatric and non-oncology cohorts, rectus femoris thickness cut-offs have ranged across different values, reflecting methodological heterogeneity. Therefore, the threshold identified in our study should be interpreted within the specific clinical and demographic context of our oncology population. Ultrasound offers a radiation-free, bedside, and repeatable method for muscle assessment, which may facilitate integration into routine oncology practice [14,17]. Importantly, ultrasonography should not be interpreted as a replacement for CT-based muscle assessment in the absence of direct comparative validation, as no head-to-head analysis between these modalities was performed in the present study.

However, the relatively lower specificity observed in ROC analyses warrants careful clinical interpretation. Lower specificity implies a higher false-positive rate, meaning that a proportion of non-sarcopenic patients may be classified as “at risk” when ultrasound-derived cut-off values are used in isolation. In routine oncology practice, this may lead to additional confirmatory testing or supportive interventions. Nevertheless, the clinical acceptability of moderate specificity depends largely on the intended use of the assessment. If ultrasonography is applied as a rapid bedside screening tool, prioritizing sensitivity may be clinically appropriate in order to minimize missed cases, particularly in oncology populations where early identification of reduced muscle reserves may influence nutritional strategies, rehabilitation planning, and treatment tolerance evaluation. In this context, false-positive classifications are less likely to result in harm if they prompt low-risk supportive measures rather than irreversible therapeutic decisions. Conversely, if ultrasound-derived thresholds are intended for confirmatory diagnosis or high-stakes clinical decision-making, higher specificity would be preferable. Therefore, the proposed cut-off values should be interpreted as exploratory and context-dependent and ideally incorporated into a stepwise diagnostic framework combining ultrasonographic findings with strength testing and clinical assessment. Future external validation in larger oncology cohorts is necessary to refine threshold selection and improve specificity.

Clinically, these findings are highly relevant. Sarcopenia is increasingly recognized as a modifiable condition that can affect treatment tolerance, quality of life, and survival in patients with cancer [27,28]. Early identification of patients at risk enables the timely implementation of nutritional support, resistance exercise programs, and individualized treatment strategies. Therefore, combining ultrasonographic muscle assessment with handgrip strength and BIA may optimize risk stratification and support personalized care, particularly in settings with limited access to advanced imaging.

Several limitations should be acknowledged. The single-center design may limit the generalizability of our findings. Ultrasonographic measurements were performed by a single trained investigator, precluding assessment of inter-observer variability. Additionally, echo intensity was not analyzed; therefore, muscle quality and intramuscular fat infiltration could not be assessed. Physical performance was not assessed; therefore, severe sarcopenia could not be determined according to EWGSOP2 criteria. Moreover, nutritional status was not formally assessed using standardized screening tools, which may have influenced muscle mass and strength parameters. In addition, skeletal muscle mass was estimated using single-frequency bioelectrical impedance analysis, which may be influenced by hydration status and fluid distribution, particularly in oncology populations where fluid imbalance is common. Furthermore, the relatively small number of patients classified as sarcopenic may limit the stability and generalizability of subgroup analyses and ROC-derived cut-off values. Given the limited number of sarcopenic cases, the statistical power to detect subgroup differences may have been reduced. In addition, multivariable regression analyses were necessarily restricted to a small number of covariates to reduce the risk of overfitting. Patients with poor general condition (ECOG performance status ≥ 3) were excluded, which may have led to underrepresentation of more functionally impaired individuals and potentially influenced the observed prevalence of sarcopenia. The imbalance in metastatic disease distribution between groups may also have confounded comparisons and limits causal interpretation. Comorbidities were not quantified using a standardized comorbidity index (e.g., Charlson Comorbidity Index), which may have limited a more comprehensive evaluation of their potential influence on sarcopenia risk. No a priori sample size or power calculation was performed, which may limit the statistical precision of the findings. Finally, the cross-sectional nature of the study does not allow evaluation of longitudinal changes in muscle mass or their direct impact on long-term clinical outcomes such as survival.

Our study also has important strengths that contribute to the literature. The prospective design and standardized measurement protocols increase data reliability and reproducibility. Most importantly, sarcopenia was evaluated using a comprehensive multimodal framework integrating functional (handgrip strength), quantitative (BIA), and morphological (ultrasound) parameters rather than relying solely on muscle mass assessment. To our knowledge, few prospective studies have examined rectus femoris muscle architecture in such detail in oncology populations within a multimodal diagnostic framework. This study is also among the limited number of prospective investigations in Turkey to examine rectus femoris muscle architecture in patients with cancer and to provide specific cut-off values. In this respect, our findings offer a tangible contribution to both local and broader literature regarding bedside sarcopenia screening in oncology practice, providing preliminary evidence for the feasibility of ultrasonographic assessment as a screening component while underscoring the need for external validation before broader clinical implementation.

## 5. Conclusions

This prospective cross-sectional study demonstrates that both sarcopenia and probable sarcopenia are highly prevalent among patients with cancer followed in routine oncology practice. A multimodal assessment strategy integrating handgrip strength, bioelectrical impedance analysis (BIA), and ultrasonographic muscle measurements provides a comprehensive, reliable, and clinically feasible model for sarcopenia screening. In particular, ultrasonographic measurements of the rectus femoris muscle—specifically muscle thickness and cross-sectional area—showed high diagnostic performance for predicting sarcopenia, independent of age and sex, supporting their role as independent markers of muscle impairment. Incorporating this morphological assessment into routine oncology care may facilitate early identification of patients at risk and enable timely nutritional and rehabilitative interventions. Overall, close monitoring of muscle health using this multimodal approach has the potential to improve treatment tolerance, preserve quality of life, and enhance clinical outcomes in patients with cancer.

## Figures and Tables

**Table 1:** Baseline demographic and clinical characteristics of the study population.

	Total Patients (*n* = 147)
**Age (years)**	60.18 ± 11.21 ^a^
**Sex, *n* (%)**	
Female	72 (49%)
Male	75 (51%)
**Height (cm)**	161.99 ± 9.35 ^a^
**Weight (kg)**	69.62 ± 12.66 ^a^
**BMI (kg/m^2^)**	26.37 ± 5.50 ^a^
**Malignancy Group, *n* (%)**	
Respiratory	35 (23.8%)
Gastrointestinal	45 (30.6%)
Breast/Gynecological	54 (36.7%)
Urological	9 (6.1%)
Other	4 (2.7%)
**Time since diagnosis (days)**	154 [58.75–462.75] ^b^
**Metastatic disease, *n* (%)**	
Yes	26 (17.7%)
No	121 (82.3%)
**Comorbidities, *n* (%)**	83 (56.5%)
**Number of comorbidities**	2 [1,2,3] ^b^

**Footnote:** ^a^: Mean ± Standard Deviation, ^b^: Median [Interquartile Range]. BMI: Body Mass Index.

**Table 2:** Assessment of Muscle Strength and BIA-derived Body Composition Parameters.

	Total Patients (*n* = 147)
**Handgrip strength (kg)**	
Dominant hand	23.28 ± 8.58 ^a^
Low muscle strength (Probable sarcopenia)	58 (39.5%)
**Body composition (BIA)**	
Total Muscle mass (kg)	46.45 ± 7.57 ^a^
SMM (kg)	24.82 ± 4.87 ^a^
ASMI (kg/m^2^)	7.08 ± 1.20 ^a^
**Sarcopenia status, *n* (%)**	
Sarcopenia	18 (12.2%)
Isolated Low Muscle Mass	7 (4.8%)
Probable Sarcopenia	40 (27.2%)
No Sarcopenia (Normal)	82 (55.8%)

**Footnote:** ^a^: Mean ± Standard Deviation. SMM: Skeletal Muscle Mass, ASMI: Appendicular Skeletal Muscle Mass Index. Probable Sarcopenia: Defined by low muscle strength (Handgrip strength < 27 kg for males, <16 kg for females). Isolated Low Muscle Mass: Normal muscle strength but low muscle mass (ASMI below the cut-off thresholds). Sarcopenia: Confirmed by the presence of both low muscle strength (Handgrip) and low muscle quantity (ASMI and BIA). ASMI Cut-off Points: Low muscle mass was defined as ASMI < 7.0 kg/m^2^ for males and <5.5 kg/m^2^ for females.

**Table 3:** Ultrasonographic Assessment of Rectus Femoris and Biceps Brachii Muscle Architecture.

	Total Patients (*n* = 147)
**Rectus femoris ultrasound (right side)**	
Muscle thickness (mm)	7.98 ± 2.20 ^a^
Cross-sectional area (cm^2^)	2.03 ± 0.60 ^a^
**Biceps brachii ultrasound (right side)**	
Muscle thickness (mm)	8.80 ± 2.33 ^a^
Cross-sectional area (cm^2^)	2.15 [1.61–2.65] ^b^

**Footnote:** ^a^: Mean ± Standard Deviation, ^b^: Median [Interquartile Range].

**Table 4:** Comparison of Demographic, Clinical, and BIA-Derived Characteristics Between Patients with and Without Sarcopenia.

	Sarcopenia (*n* = 18)	Non-Sarcopenia (*n* = 129)	*p*-Value
**Age, years (mean ± SD)**	68.50 ± 7.64 ^a^	59.02 ± 11.16 ^a^	0.001 ^c,^*
**Sex, *n* (%)**			0.001 ^d,^*
Female	2 (11.1%)	70 (54.3%)	
Male	16 (88.9%)	59 (45.7%)	
**BMI (kg/m^2^)**	27.48 ± 4.85 ^a^	26.57 ± 5.23 ^a^	0.486 ^c^
**Malignancy Group, *n* (%)**			0.003 ^d,^*
Respiratory	9 (50%) ⱡ	26 (20.2%)	
Gastrointestinal	7 (38.9%)	38 (29.5%)	
Breast/Gynecological	1 (5.6%)	53 (41.1%) ⱡ	
Urological	0 (0%)	9 (7%)	
Other	1 (5.6%)	3 (2.3%)	
**Time since diagnosis (days)**	167 [34–536.50] ^b^	153 [61.50–446.50] ^b^	0.838 ^e^
**Metastatic disease, *n* (%)**	2 (11.1%)	24 (18.6%)	0.741 ^f^
**Comorbidities, *n* (%)**	13 (72.2%)	70 (54.3%)	0.150 ^d^
**Number of comorbidities**	3 [1.50–3] ^b^	2 [1,2,3] ^b^	0.737 ^e^
**Handgrip strength (kg)**			
Dominant hand	15.79 ± 6.01 ^a^	24.32 ± 8.38 ^a^	<0.001 ^c,^*
Low muscle strength	18 (100%)	40 (31%)	<0.001 ^d,^*
**Body composition (BIA)**			
Total muscle mass (kg)	42.81 ± 4.29 ^a^	46.96 ± 7.80 ^a^	0.002 ^c,^*
SMM (kg)	21.81 ± 3.20 ^a^	25.24 ± 4.93 ^a^	<0.001 ^c,^*
ASMI (kg/m^2^)	5.96 ± 0.64 ^a^	7.23 ± 1.18 ^a^	<0.001 ^c,^*

**Footnote:** ^a^: Mean ± Standard Deviation, ^b^: Median [Interquartile Range], ^c^: Independent *T* Test, ^d^: Chi-Square Test, ^e^: Mann–Whitney U Test, ^f^: Fisher’s Exact Test. ⱡ: Indicates a statistically significant difference between groups. *: Statistically significant (*p* < 0.05). BMI: Body Mass Index, SMM: Skeletal Muscle Mass, ASMI: Appendicular Skeletal Muscle Mass Index.

**Table 5:** Comparison of Ultrasonographic Parameters Between Patients with and Without Sarcopenia.

	Sarcopenia (*n* = 18)	Non-Sarcopenia (*n* = 129)	*p*-Value
**Rectus femoris ultrasound (right side)**			
Muscle thickness (mm)	6.40 ± 1.42 ^a^	8.19 ± 2.21 ^a^	0.001 ^c,^*
Cross-sectional area (cm^2^)	1.72 ± 0.41 ^a^	2.08 ± 0.61 ^a^	0.003 ^c,^*
**Biceps brachii ultrasound (right side)**			
Muscle thickness (mm)	7.88 ± 2.39 ^a^	8.92 ± 2.30 ^a^	0.076 ^c^
Cross-sectional area (cm^2^)	1.88 [1.38–2.59] ^b^	2.15 [1.67–2.65] ^b^	0.260 ^d^

**Footnote:** ^a^: Mean ± Standard Deviation, ^b^: Median [Interquartile Range], ^c^: Independent *T* Test, ^d^: Mann–Whitney U Test. *: Statistically significant (*p* < 0.05).

**Table 6:** Correlation between BIA-derived muscle parameters, handgrip strength, and ultrasonographic measurements.

	Handgrip Strength		Total Muscle Mass		SMM		ASMI	
	r	*p*	r	*p*	r	*p*	r	*p*
**Rectus femoris**								
MT ^a^	0.371	<0.001 *	0.253	0.002 *	0.156	0.060	0.195	0.018 *
CSA ^a^	0.414	<0.001 *	0.284	<0.001 *	0.135	0.104	0.180	0.029 *
**Biceps brachii**								
MT ^a^	0.298	<0.001 *	0.241	0.003 *	0.108	0.192	0.117	**0.159**
CSA ^b^	0.377	<0.001 *	0.217	0.008 *	0.123	0.139	0.172	0.038 *

**Footnote:** Pearson correlation analysis was used for parameters marked with a, and Spearman correlation analysis was used for parameters marked with b. r: indicates the correlation coefficient. Statistically significant correlations are indicated with an asterisk (*p* < 0.05). SMM: Skeletal Muscle Mass, ASMI: Appendicular Skeletal Muscle Mass Index, MT: Muscle Thickness, CSA: Cross-Sectional Area.

**Table 7:** Logistic Regression Analysis of Factors Associated with Sarcopenia.

	B	S.E.	Wald	OR (%95 CI)	*p*
***Model 1***					
Age (years)	0.068	0.034	4.131	1.071 (1.002–1.143)	0.042 *
Sex (Male)	2.231	0.804	7.701	9.313 (1.926–45.036)	0.006 *
RF-MT (mm)	−0.489	0.174	7.918	0.613 (0.436–0.862)	0.005 *
***Model 2***					
Age (years)	0.078	0.033	5.473	1.081 (1.013–1.154)	0.019 *
Sex (Male)	2.203	0.801	7.572	9.056 (1.885–43.504)	0.006 *
RF-CSA (cm^2^)	−1.140	0.544	4.391	0.320 (0.110–0.929)	0.036 *

**Footnote:** Multivariate logistic regression analyses were adjusted for age and sex. The Hosmer–Lemeshow test indicated good model fit for both models (Model 1: *p* = 0.922; Model 2: *p* = 0.867). OR: Odds Ratio, CI: Confidence Interval, RF-MT: Rectus Femoris Muscle Thickness, RF-CSA: Rectus Femoris Cross-Sectional Area. *: Statistically significant (*p* < 0.05).

**Table 8:** Diagnostic Performance of Ultrasonographic Parameters in Detecting Sarcopenia.

Parameters	AUC (95% CI)	Cut-Off	Sensitivity (%)	Specificity (%)	*p*
RF-MT	0.752 (0.650–0.853)	≤7.59 mm	83.3	61.2	0.001 *
RF-CSA	0.681 (0.571–0.791)	≤2.07 cm^2^	88.9	51.2	0.013 *

**Footnote:** Smaller values of RF-MT and RF-CSA indicate a higher likelihood of sarcopenia. *: Statistically significant (*p* < 0.05). AUC: Area Under the Curve, RF-MT: Rectus Femoris Muscle Thickness, RF-CSA: Rectus Femoris Cross-Sectional Area.

## Data Availability

Dataset available on request from the authors.

## References

[1] Cruz-Jentoft A.J., Bahat G., Bauer J., Boirie Y., Bruyère O., Cederholm T., Cooper C., Landi F., Rolland Y., Sayer A.A. (2019). Sarcopenia: Revised European consensus on definition and diagnosis. Age Ageing.

[2] Bauer J., Morley J.E., Schols A.M.W.J., Ferrucci L., Cruz-Jentoft A.J., Dent E., Baracos V.E., Crawford J.A., Doehner W., Heymsfield S.B. (2019). Sarcopenia: A Time for Action. An SCWD Position Paper. J. Cachexia Sarcopenia Muscle.

[3] Shachar S.S., Williams G.R., Muss H.B., Nishijima T.F. (2016). Prognostic value of sarcopenia in adults with solid tumours: A meta-analysis and systematic review. Eur. J. Cancer.

[4] Pamoukdjian F., Bouillet T., Lévy V., Soussan M., Zelek L., Paillaud E. (2018). Prevalence and predictive value of pre-therapeutic sarcopenia in cancer patients: A systematic review. Clin. Nutr..

[5] Prado C.M., Lieffers J.R., McCargar L.J., Reiman T., Sawyer M.B., Martin L., Baracos V.E. (2008). Prevalence and clinical implications of sarcopenic obesity in patients with solid tumours of the respiratory and gastrointestinal tracts: A population-based study. Lancet Oncol..

[6] Aleixo G.F., Shachar S.S., Nyrop K.A., Muss H.B., Battaglini C.L., Williams G.R. (2019). Bioelectrical Impedance Analysis for the Assessment of Sarcopenia in Patients with Cancer: A Systematic Review. Oncologist.

[7] Saraf A., He J., Shin K.-Y., Weiss J., Awad M.M., Gainor J., Kann B.H., Christiani D.C., Aerts H.J., Mak R.H. (2025). Association of Sarcopenia with Toxicity and Survival in Patients with Lung Cancer, a Multi-Institutional Study with External Dataset Validation. Clin. Lung Cancer.

[8] Kyle U.G., Bosaeus I., De Lorenzo A.D., Deurenberg P., Elia M., Gómez J.M., Heitmann B.L., Kent-Smith L., Melchior J.-C., Pirlich M. (2004). Bioelectrical impedance analysis—Part I: Review of principles and methods. Clin. Nutr..

[9] Mourtzakis M., Prado C.M., Lieffers J.R., Reiman T., McCargar L.J., Baracos V.E. (2008). A practical and precise approach to quantification of body composition in cancer patients using computed tomography images acquired during routine care. Appl. Physiol. Nutr. Metab..

[10] Baracos V.E., Martin L., Korc M., Guttridge D.C., Fearon K.C.H. (2018). Cancer-associated cachexia. Nat. Rev. Dis. Primers.

[11] Chen L.-K., Woo J., Assantachai P., Auyeung T.-W., Chou M.-Y., Iijima K., Jang H.C., Kang L., Kim M., Kim S. (2020). Asian Working Group for Sarcopenia: 2019 Consensus Update on Sarcopenia Diagnosis and Treatment. J. Am. Med. Dir. Assoc..

[12] Prior-Sánchez I., Herrera-Martínez A.D., Zarco-Martín M.T., Fernández-Jiménez R., Gonzalo-Marín M., Muñoz-Garach A., Vilchez-López F.J., Cayón-Blanco M., Villarrubia-Pozo A., Muñoz-Jiménez C. (2024). Prognostic value of bioelectrical impedance analysis in head and neck cancer patients undergoing radiotherapy: A VALOR^®^ study. Front. Nutr..

[13] Nijholt W., Scafoglieri A., Jager-Wittenaar H., Hobbelen J.S., van der Schans C.P. (2017). The reliability and validity of ultrasound to quantify muscles in older adults: A systematic review. J. Cachex-Sarcopenia Muscle.

[14] Perkisas S., Bastijns S., Baudry S., Bauer J., Beaudart C., Beckwée D., Cruz-Jentoft A., Gasowski J., Hobbelen H., Jager-Wittenaar H. (2021). Application of ultrasound for muscle assessment in sarcopenia: 2020 SARCUS update. Eur. Geriatr. Med..

[15] Isaka M., Sugimoto K., Akasaka H., Yasunobe Y., Takahashi T., Xie K., Onishi Y., Yoshida S., Minami T., Yamamoto K. (2022). The Muscle Thickness Assessment Using Ultrasonography is a Useful Alternative to Skeletal Muscle Mass by Bioelectrical Impedance Analysis. Clin. Interv. Aging.

[16] Esposto G., Borriello R., Galasso L., Termite F., Mignini I., Cerrito L., Ainora M.E., Gasbarrini A., Zocco M.A. (2024). Ultrasound Evaluation of Sarcopenia in Patients with Hepatocellular Carcinoma: A Faster and Easier Way to Detect Patients at Risk. Diagnostics.

[17] Ticinesi A., Meschi T., Narici M.V., Lauretani F., Maggio M. (2017). Muscle Ultrasound and Sarcopenia in Older Individuals: A Clinical Perspective. J. Am. Med. Dir. Assoc..

[18] Abe T., Loenneke J.P., Young K.C., Thiebaud R.S., Nahar V.K., Hollaway K.M., Stover C.D., Ford M.A., Bass M.A., Loftin M. (2015). Validity of Ultrasound Prediction Equations for Total and Regional Muscularity in Middle-aged and Older Men and Women. Ultrasound Med. Biol..

[19] Mathiowetz V., Weber K., Volland G., Kashman N. (1984). Reliability and validity of grip and pinch strength evaluations. J. Hand Surg..

[20] Janssen I., Heymsfield S.B., Baumgartner R.N., Ross R. (2000). Estimation of skeletal muscle mass by bioelectrical impedance analysis. J. Appl. Physiol..

[21] Alemán-Mateo H., Valenzuela R.E.R. (2014). Skeletal Muscle Mass Indices in Healthy Young Mexican Adults Aged 20–40 Years: Implications for Diagnoses of Sarcopenia in the Elderly Population. Sci. World J..

[22] Proctor D.N., O’brien P.C., Atkinson E.J., Nair K.S. (1999). Comparison of techniques to estimate total body skeletal muscle mass in people of different age groups. Am. J. Physiol. Metab..

[23] Hansen R.D., Raja C., Aslani A., Smith R.C., Allen B.J. (1999). Determination of skeletal muscle and fat-free mass by nuclear and dual-energy X-ray absorptiometry methods in men and women aged 51–84 y. Am. J. Clin. Nutr..

[24] López Jiménez E., Neira Álvarez M., Ramírez Martín R., Alonso Bouzón C., Amor Andrés M.S., Bermejo Boixareu C., Brañas F., Menéndez Colino R., Arias Muñana E., Checa López M. (2023). Sarcopenia measured by ultrasound in hospitalized older adults (ECOSARC): Multi-centre, prospective observational study protocol. BMC Geriatr..

[25] Matsui Y., Takemura M., Suzuki Y., Watanabe T., Maeda K., Satake S., Arai H. (2024). Evaluation of quadriceps muscle cross-sectional area using an ultrasonic diagnostic equipment with a wide field of view. PLoS ONE.

[26] Li S., Xu K., Wang A., Qin C., Hua N., Ling X., Xu L., He C., Zhou S., Chen J. (2025). Determination of Ultrasound Reference Values for Diagnosing Low Muscle Mass in Older Chinese Adults. J. Cachex-Sarcopenia Muscle.

[27] Fearon K.C., Glass D.J., Guttridge D.C. (2012). Cancer Cachexia: Mediators, Signaling, and Metabolic Pathways. Cell Metab..

[28] Prado C.M., Purcell S.A., Alish C., Pereira S.L., Deutz N.E., Heyland D.K., Goodpaster B.H., Tappenden K.A., Heymsfield S.B. (2018). Implications of low muscle mass across the continuum of care: A narrative review. Ann. Med..

[29] Cruz-Jentoft A.J., Sayer A.A. (2019). Sarcopenia. Lancet.

[30] Cesari M., Landi F., Vellas B., Bernabei R., Marzetti E. (2014). Sarcopenia and Physical Frailty: Two Sides of the Same Coin. Front. Aging Neurosci..

[31] Kilgour R.D., Vigano A., Trutschnigg B., Hornby L., Lucar E., Bacon S.L., Morais J.A. (2010). Cancer-related fatigue: The impact of skeletal muscle mass and strength in patients with advanced cancer. J. Cachex-Sarcopenia Muscle.

[32] Reinders I., Visser M., Schaap L. (2017). Body weight and body composition in old age and their relationship with frailty. Curr. Opin. Clin. Nutr. Metab. Care.

[33] Perkisas S., De Cock A.-M., Verhoeven V., Vandewoude M. (2017). Intramuscular Adipose Tissue and the Functional Components of Sarcopenia in Hospitalized Geriatric Patients. Geriatrics.

[34] Huang Y., Liu S., Muo C., Lai C., Chang C. (2024). Male Sex and Ageing Are Independent Risk Factors for Sarcopenia Stage in Patients with Chronic Kidney Disease Not Yet on Dialysis. J. Cachex-Sarcopenia Muscle.

[35] Yuan S., Larsson S.C. (2023). Epidemiology of sarcopenia: Prevalence, risk factors, and consequences. Metabolism.

[36] Beaudart C., Zaaria M., Pasleau F., Reginster J.-Y., Bruyère O. (2017). Health Outcomes of Sarcopenia: A Systematic Review and Meta-Analysis. PLoS ONE.

